# Supplementary material for: Active optical phased array integrated within a micro-cantilever
Source: Commun Eng. 2024 Jun 4;3:76. doi: 10.1038/s44172-024-00224-1 (PMC11150254; doi:10.1038/s44172-024-00224-1)
Supplement: Supplementary file 2 — Supplementary Information [file 44172_2024_224_MOESM2_ESM.pdf]

Active optical phased array integrated within a micro-cantilever

SYLVAIN GUERBER, DAIVID FOWLER*, LAURENT MOLLARD, CHRISTEL DIEPPEDALE, GWENAEL LE RHUN, ANTOINE HAMELIN, JONATHAN FAUGIER-TOVAR, KIM ABDOUL-CARIME

Univ. Grenoble Alpes, CEA, LETI, F38000 Grenoble, France
**daivid.fowler@cea.fr*

SUPPLEMENTARY INFORMATION

Supplementary note 1

OPA and cantilever synchronous driving for resonant beam scanning demonstration

The architecture of the OPA characterization bench used in this work has been presented in previous publications[1,2]. It is based on a standard 300 mm probing station (Cascade Elite 300) typically used in the microelectronic industry for wafer-level circuit testing. In order to characterize the scanning properties of the OPA circuit while driving the cantilever in a resonant state, a specific control system has been used. A schematic view of the setup is presented in Supplementary figure 1(a). The cantilever PZT actuator is driven by a sinusoidal signal coming from a 3 channel Arbitrary Waveform Generator (AWG, Spectrum Instrumentation DN2.656-16). The light from a pulsed laser (Aerodiode Shaper with Fabry-Perot laser diode) is coupled to an optical fiber and passes through a variable attenuator (not shown) and a fiber polarization controller (FPC) before being injected at the OPA input using a grating coupler. The emission of a light pulse from the laser is controlled by an external trigger signal generated by the AWG. The 16 phase shifters (thermo-optical modulators) of the OPA are individually controlled by a custom made electronic board (OPA control board). In order to limit the power dissipation due to the heat generated by the thermo-optical modulators, the output of the control board is operated in a pulsed regime where the 16 electrical channels are being enabled/disabled by a periodic square signal generated by the AWG. Due to space constraints, the OPA far field emission is imaged directly on the bare sensor of a CCD camera (Allied Vision Prosilica GT 6600) with no intermediate lenses. All of the instruments are controlled by a master computer and Python programs.

In order to take an instantaneous / frozen image of the emitted beam while the cantilever is dynamically actuated, we rely on the Strobe effect by synchronizing all the control signals emitted by the AWG as presented in Supplementary figure 1(b). A sinusoidal signal drives the cantilever at its resonant frequency $f_{cant} = 1.52\, kHz$. The laser pulses are generated with a repetition rate that correspond to an integer lower multiple (60) of the cantilever driving frequency $f_{las\_heat} = f_{cant}/60 = 25.33\, Hz$, thus the cantilever is always in the same position when a light pulse is sent to the OPA. In addition, the emission of the laser pulses must be synchronized with the activation of the phase modulators, so that the emitted light will be shaped by the OPA. Thus, the square signal that drives the activation of the OPA modulators have the same frequency as the laser driving signal f_{las_heat}. The electrical pulse duration is set to $\tau_c \approx 30\ \mu s$, and the laser pulse is sent with a delay $\Delta_{las} \approx 20\ \mu s$ that is slightly larger than the thermo-optical modulator response time (corresponding to a cutoff frequency of ~50 kHz). Finally, the imaging camera (that cannot be synchronized, no external trigger input) integration time is set to a value corresponding to the laser

repetition rate, thus avoiding image blinking $Integ_{camera} = 1/f_{las\_heat} = 39474\ \mu s$. Thus, only a single laser pulse is exposed on each picture captured by the camera. The laser output power is then adapted using the variable attenuator to avoid saturation of the camera.

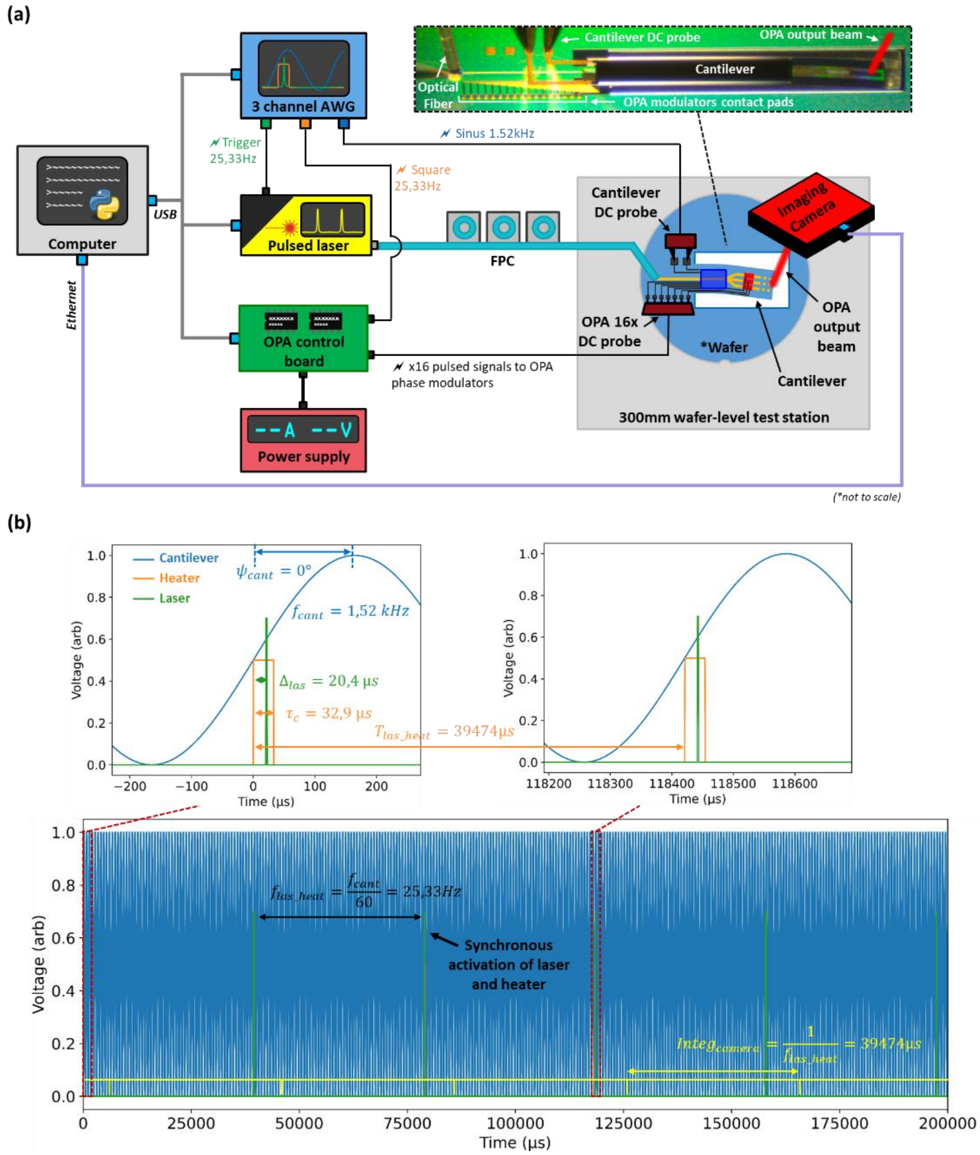

Supplementary figure 1. Synchronous measurement method (a) Schematic of the optical phased array (OPA) and cantilever synchronous driving system. (b) Temporal view of the different synchronization signals generated by the AWG.

Therefore, by tuning the phase ψ_{cant} of the sinusoidal driving signal (that corresponds to a relative time shift with the laser/heater activation), we are able to capture an image with the cantilever in a certain position. This technique was used to acquire the composite image presented in supplementary figure 2, demonstrating the 2D scanning capability of the cantilever-OPA. The cantilever is excited at its resonant frequency and the driving signal phase is set to a certain value, fixing the beam output angle on the θ axis. Then, the OPA is operated to scan the beam on the ϕ axis and images are taken at some discrete angles (here +/-10°, 2° steps). Then the driving signal phase is changed (here from 160 to 300°), and the OPA scanning process is repeated. The phase value are chosen to fully map the cantilever scanning range. Then, all the acquired images are stacked (individual pixel summation) in order to get the composite image.

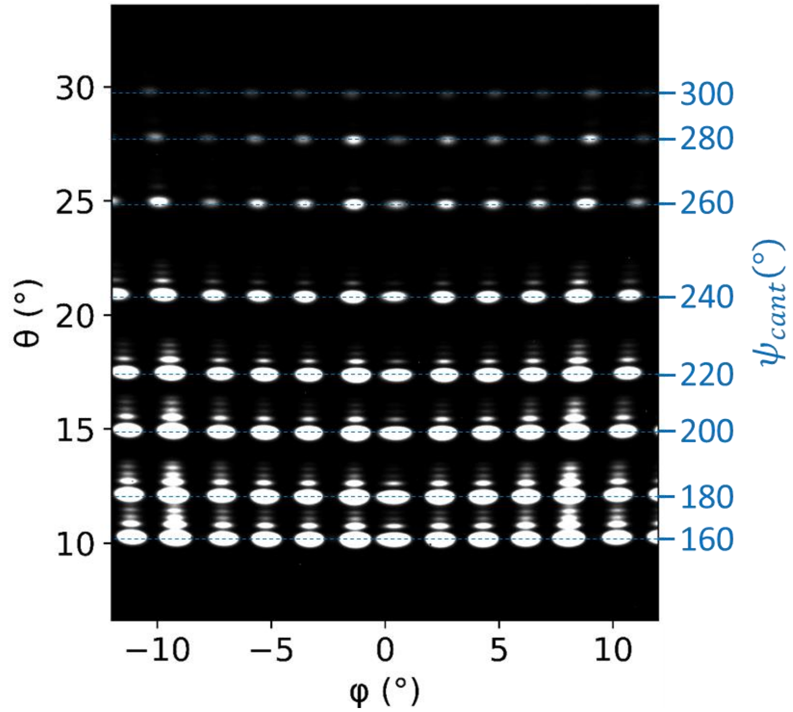

Supplementary figure 2. Composite image of the optical phased array output configured for various main beam emission in φ and θ while the cantilever is excited at its resonance frequency fcant = 1.52 kHz.

Supplementary note 2

PZT electromechanical properties

Electrical characterization of the PZT layer showed that the maximum permittivity is around 1100 and the maximum polarization is 37 µC/cm² at an applied voltage of 20 V. Supplementary figure 3 shows the hysteresis loop of the transverse piezoelectric coefficient, $d_{31}(V)$, derived using the method in [3] obtained at the end of the process. The maximum piezoelectric coefficient is d_{31max} ~ 130pm/V.

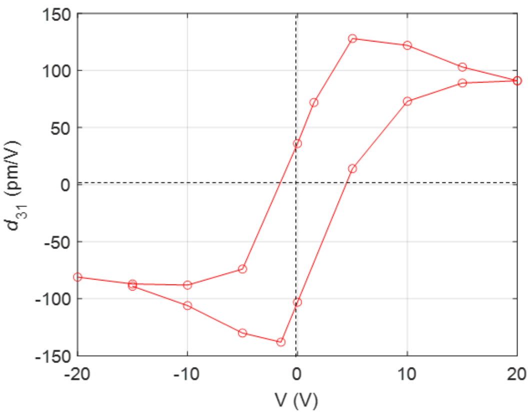

Supplementary figure 3 Transverse piezoelectric coefficient d_{31max}(V) hysteresis loop obtained at the end of the lead zirconate titanate (PZT) transfer process

The hysteresis of the PZT actuator, which is expected for ferroelectric PZT based actuator, can be seen in the displacement of the cantilever in the quasi-static regime. The characteristic S-curve of angular displacement versus applied voltage for an identically fabricated (but not identically dimensioned) cantilever to that used in the main text is shown in supplementary figure 4.

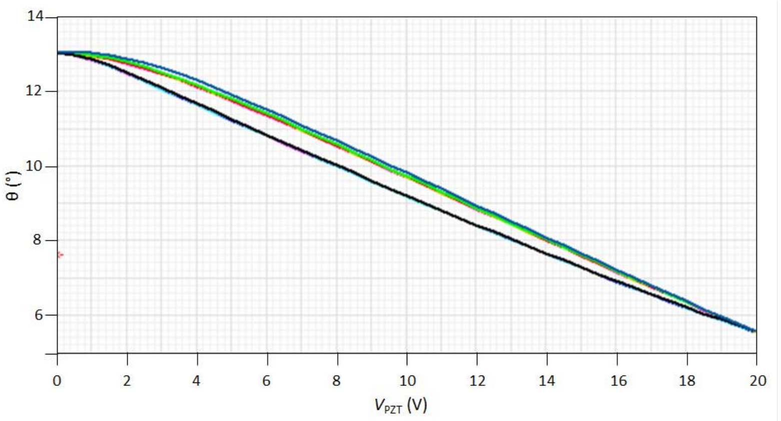

Supplementary figure 4 Cantilever angular displacement versus applied DC voltage showing hysteresis effect. The different colored lines represent consecutive actuation cycles

Supplementary note 3

Long exposure acquisition to measure the maximum beam scanning amplitude in the AC regime

As presented in

Supplementary figure 5(a), we used some long exposure images ($Integ_{camera} \gg 1/f_{cant}$) in order to measure the minimum θ_{min} and maximum θ_{max} deflection of the output beam on the θ axis. Light is injected inside the OPA, the cantilever is excited with a sinusoidal signal corresponding to its resonant frequency ($f_{cant} = 1.52kHz$) and a long exposure image is taken. The extracted beam scanning amplitude as a function of the cantilever driving signal voltage amplitude is presented

Supplementary figure 5(b). First (region I), both θ_{min} and θ_{max} increase/decrease linearly with the driving signal voltage amplitude (expected behavior). However for a voltage >6V (region II), the measured θ_{max} stops increasing and is fixed at ~28° (while θ_{min} continues to decrease linearly). As shown on the drawings I and II in supplementary figure 5(c), this behavior is due to the output beam being obscured by the wall of the cantilever cavity (insufficient spacing between the cantilever tip and the end of the cavity), which can be understood by viewing the moving cantilever in supplementary video 1. This issue can be solved simply by etching a wider area in front of the cantilever. This also explains why the output beam intensity decreases with increasing θ angle (see supplementary figure 5), the light emitted by the OPA antennas is gradually shaded while the cantilever is bending towards the substrate (increasing θ). Thus, the maximum measured beam scanning amplitude is $\Delta\theta \approx 26°$ (@ 16V). Using projection of the linear fit on the first part of the θ_{max} curve, we can compute the "hidden θ_{max}" which gives a maximum beam scanning amplitude $\Delta\theta \approx 42°$. Finally, when the voltage is further increased (>16V), catastrophic failure occurred, with the dielectric breakdown of the PZT actuator (region III supplementary figure 5(b)). The origin of this damage remains under investigation, but it is likely that doping with La or Nb, can help increase resistance to dielectric breakdown of the PZT layer[4].

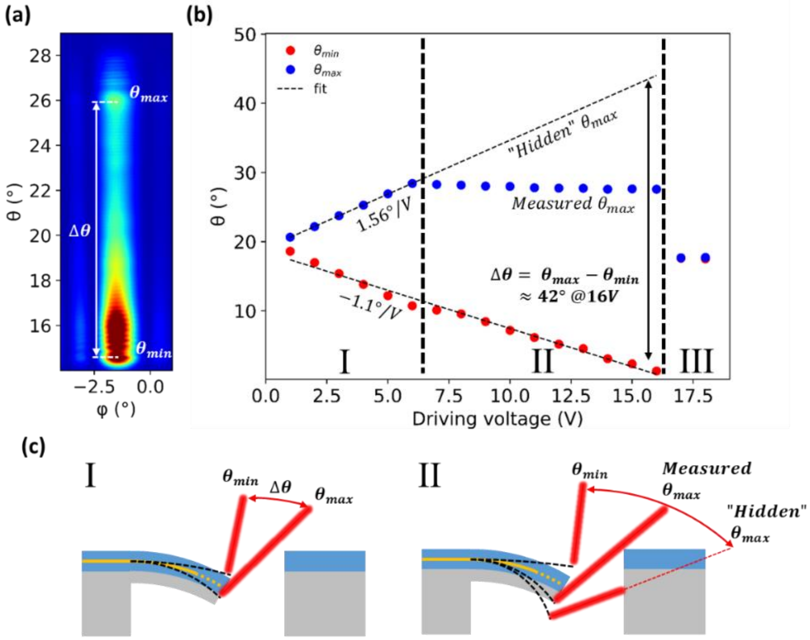

Supplementary figure 5, Measurement of high deflection angles (a) Long exposure image of the optical phased array output beam while the cantilever is moving due to the electrical excitation. (b) Measured maximum beam scanning amplitude on the cantilever (θ) axis.

Supplementary References

1. Guerber, S. *et al.* Development, calibration and characterization of silicon photonics based optical phased arrays. in *Smart Photonic and Optoelectronic Integrated Circuits XXIII* vol. 11690 22–29 (SPIE, 2021).
2. Guerber, S. *et al.* Wafer-level calibration of large-scale integrated optical phased arrays. *Opt. Express, OE* **30**, 35246–35255 (2022).
3. Nakajima, M., Okamoto, S., Nakaki, H., Yamada, T. & Funakubo, H. Enhancement of piezoelectric response in (100)/(001) oriented tetragonal Pb(Zr, Ti)O3 films by controlling tetragonality and volume fraction of the (001) orientation. *Journal of Applied Physics* **109**, 091601 (2011).
4. Barrett, N., Gueye, I., Rhun, G. L., Renault, O. & Defay, E. Unexpected band-bending of donor-doped PbZr0.52Ti0.48O3 films. *Thin Solid Films* **715**, 138423 (2020).
