# Supplementary material for: Active optical phased array integrated within a micro-cantilever
Source: Commun Eng. 2024 Jun 4;3:76. doi: 10.1038/s44172-024-00224-1 (PMC11150254; doi:10.1038/s44172-024-00224-1)
Supplement: Supplementary file 3 — Description of Additional Supplementary Files [file 44172_2024_224_MOESM3_ESM.pdf]

Description of Additional Supplementary Files

File name: Supplementary Video 1

File Description: Cantilever motion in the completed device in quasi-static operation.
