# Supplementary figures and images for: Active optical phased array integrated within a micro-cantilever
Source: Commun Eng. 2024 Jun 4;3:76. doi: 10.1038/s44172-024-00224-1 (PMC11150254; doi:10.1038/s44172-024-00224-1)

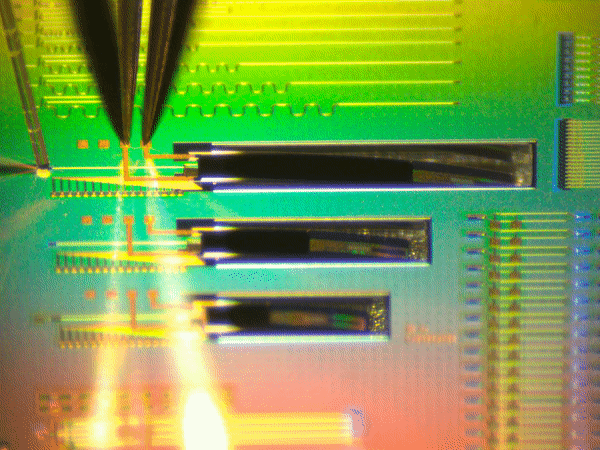

Supplement: Supplementary file 4 — supplementary movie 1 [file 44172_2024_224_MOESM4_ESM.gif]
